# Ending overly broad HIV criminalization: Canadian scientists and clinicians stand for justice

**DOI:** 10.7448/IAS.18.1.20126

**Published:** 2015-07-17

**Authors:** Cécile Kazatchkine, Edwin Bernard, Patrick Eba

**Affiliations:** 1 Canadian HIV/AIDS Legal Network, Toronto, ON, Canada; 2 HIV Justice Network, Brighton, UK; 3 UNAIDS, Geneva, Switzerland

**Keywords:** HIV, criminal law, scientists, Canadian consensus statement, transmission risk, disclosure

## Abstract

In Canada, people living with HIV who do not disclose their HIV status prior to sexual acts risk prosecution for aggravated sexual assault even if they have sex with a condom or while having a low (or undetectable) viral load, they had no intent to transmit HIV, and no transmission occurred. In 2013, six distinguished Canadian HIV scientists and clinicians took ground-breaking action to advance justice by co-authoring the “Canadian consensus statement on HIV and its transmission in the context of the criminal law.” This effort was born out of the belief that the application of criminal law to HIV non-disclosure was being driven by a poor appreciation of the science of HIV. More than 75 HIV scientists and clinicians Canada-wide have now endorsed the statement, agreeing that “[they] have a professional and ethical responsibility to assist those in the criminal justice system to understand and interpret current medical and scientific evidence regarding HIV.” As some 61 countries have adopted laws that specifically allow for HIV criminalization, and prosecutions for HIV non-disclosure, exposure and transmission have been reported in at least 49 countries, the authors hope that others around the world will take similar action.

In 2012, the Supreme Court of Canada ruled that people living with HIV can be imprisoned for having sex with a condom *or* while having a low (or undetectable) viral load if they have not first disclosed that they are HIV-positive. According to the court, people living with HIV have a legal duty to disclose their status to a partner if the sex poses a “realistic possibility of HIV transmission” [1]. The court said that “as a general matter,” a realistic possibility of HIV transmission is negated where 1) the accused has a low viral load *and* 2) a condom is used during the sexual act [1]. Otherwise, in Canada, a person is at risk of prosecution for aggravated sexual assault even if they had no intent to transmit HIV and no transmission occurred.

The ruling left limited legal recourse to those working to end unjust prosecutions [2–4]. Yet, several months after the decision's release, six distinguished Canadian HIV scientists and clinicians took ground-breaking action to advance justice by co-authoring the “Canadian consensus statement on HIV and its transmission in the context of the criminal law” [“the (consensus) statement”] [5].

This effort was born out of the belief that the application of criminal law to HIV non-disclosure was being driven by a poor appreciation of the science of both HIV as a chronic manageable disease and its risks of transmission.

Aimed squarely at the justice system and informed by HIV community, public health and human rights concerns, the consensus statement was based on a review of the most relevant, reliable and up-to-date medical and scientific evidence. It sets out in clear, concise and understandable terms a collective expert opinion about HIV sexual transmission, transmission associated with biting and spitting, and HIV as a chronic manageable condition.

One key area of consensus described in the statement is that, contrary to the Supreme Court's interpretation, both vaginal and anal sex with a condom pose a *negligible* possibility of transmission, whether or not the HIV-positive partner has a low viral load. In fact, “[w]hen used correctly and no breakage occurs, condoms are 100% effective at stopping the transmission of HIV” [5]. In addition, the statement notes that “evidence suggests that the possibility of sexual transmission of HIV from an HIV-positive individual to an HIV-negative individual via unprotected [i.e., condomless] vaginal intercourse approaches zero when the HIV-positive individual is taking antiretroviral therapy and has an undetectable viral load” [5].

The statement is consistent with UNAIDS’ 2013 guidance, which clearly stipulates that the criminal law should never be applied in cases of alleged HIV non-disclosure when there is no intent to harm, but especially when the risks of transmission are not significant (e.g., when a condom is used consistently, the HIV-positive person is on effective treatment or has a low viral load, or in cases of oral sex) [6].

Importantly, the consensus statement does not employ the risk categories traditionally used in public health, which often describe activities from “high risk to no risk.” Knowing that these descriptors can contribute to an exaggerated sense of risk when taken out of context, Canadian experts described the per-act possibility of HIV transmission through sex, biting or spitting along a continuum from “low possibility to negligible possibility, to no possibility of transmission” [5]. These unique categories better reflect that so-called “risky” activities “carry a per-act possibility of transmission that is much lower than is often commonly believed” [5]. Also noteworthy is that the conclusions in the statement expressing scientific consensus are strong and relatively free of conditions.

In 2008, Swiss HIV experts released a statement titled “HIV-positive individuals not suffering from any other STI and adhering to an effective antiretroviral treatment do not transmit HIV sexually” [7]. Although the statement had some success in terms of influencing criminal law around perceived HIV exposure [8], its bold conclusions raised concerns in the international scientific community, as some considered them premature [9]. Others worried about their implications on condom-based safer sex messages [10]. Mindful of this controversy, the Canadian authors emphasized that their statement is meant to inform the criminal justice system and is intended neither for public health messaging nor for the development and delivery of HIV policy and programmes. They also relied on new evidence that has since confirmed the dramatic impact of treatment on viral load and HIV transmission risk [11, 12].

More than 75 HIV scientists and clinicians Canada-wide have now endorsed the statement, agreeing that “[they] have a professional and ethical responsibility to assist those in the criminal justice system to understand and interpret current medical and scientific evidence regarding HIV” [5].

We hope that other groups around the world will take similar action as overly broad HIV criminalization is not unique to Canada. It is estimated that some 61 countries have adopted laws that specifically allow for HIV criminalization, while prosecutions for HIV non-disclosure, exposure and transmission have been reported in at least 49 countries [13]. UNAIDS, UNDP, the UN Special Rapporteur on the right to health, and the Global Commission on HIV and the Law have all urged that the criminal law be limited to exceptional cases of intentional transmission [6, 14–16]. But in many jurisdictions, perceived or potential exposure, regardless of intent or actual transmission of HIV, is sufficient to establish a criminal offence [17].

Scientific evidence, while not a panacea, can influence positive change. In 2013, based on medical evidence, a trial judge in Canada acquitted a man with an undetectable viral load who had condomless sex without disclosing his status [18]. At the same time, Swedish scientists produced a consensus statement on HIV transmission that has since been recognized by the courts in that country [19]. In Switzerland, the Swiss statement has supported successful law reform [20]. In 2011, Denmark suspended the only HIV-specific criminal law in Western Europe due to improved understanding of HIV-related risks and harms [15]. And in the United States, where most prosecutions occur [17], science played a crucial role in both Iowa's HIV-specific criminal law reform [21] and a recent ruling by the Supreme Court of Iowa [22], with the expectation of more changes to follow given federal recognition for the need to modernize such laws based on science [23].

Ensuring that criminal laws and proceedings employ the best available scientific evidence relating to HIV is critical in achieving justice whilst combating discrimination against people living with HIV. Scientists and clinicians are central to this process, as is the bold, undeniable language provided in the Canadian consensus statement.
